# Percutaneous trans-ulnar versus trans-radial arterial approach for coronary angiography and angioplasty, a preliminary experience at an Egyptian cardiology center

**DOI:** 10.1186/s43044-020-00089-7

**Published:** 2020-09-11

**Authors:** Mohammad Shafiq, Hesham Boushra Mahmoud, Malak Lamie Fanous

**Affiliations:** 1 Cardiology Department, Faculty of Medicine, Beni-Suef University, Beni Suef, Egypt; 2 Cardiology Department, Sohag Heart and GIT Specialized Center, Sohag, Egypt

**Keywords:** Ulnar artery, Coronary angiography, Approach, Egyptian

## Abstract

**Background:**

Trans-ulnar approach was proposed primarily for elective procedures in patients not suitable for trans-radial approach that was introduced two decades ago. The trans-ulnar approach is as safe and effective as the trans-radial approach for coronary angiography and intervention.

**Aim:**

This study’s aim was to assess the feasibility and safety of the trans-ulnar approach in coronary procedures as a preliminary experience for operators experienced in trans-radial approach with no/minimal trans-ulnar approach experience at an Egyptian center.

**Results:**

Vascular access in 120 patients was selected randomly for coronary angiography and angioplasty—80 through radial and 40 through ulnar approach. Patients were examined for local complications and Doppler evaluation to both radial and ulnar arteries a day after the procedure was done. Ulnar approach success was 82.5% versus 93.7% in the radial group; failure of ulnar artery puncture was the only cause of crossover in the ulnar group, while occurrence of persistent spasm was the leading cause of crossover in the radial group followed by radial artery tortuosity. The procedure time of coronary angiography and percutaneous coronary intervention of the ulnar group was significantly higher than that of the radial group (*P* value = 0.011 and 0.034, respectively). The mean caliber of the right ulnar artery was 2.45 ± 0.38, slightly larger than that of the radial artery 2.33 ± 0.38 at the level of the wrist, but this difference was statistically non-significant.

**Conclusion:**

Our study demonstrated that ulnar access with experienced radial operators and in our patients is a safe and practical approach for coronary angiography or angioplasty, without any major complications. Bearing in mind its high success rate, it can be used when a radial artery is not useful for the catheterization or as a default approach on the expense of slightly longer procedural time.

## Background

Trans-radial approach (TRA) PCI was introduced two decades ago [1]. Trans-ulnar approach had been proposed for elective procedures in patients not suitable for trans-radial approach. The trans-ulnar approach is as safe and effective as the trans-radial approach for coronary angiography and intervention. It is an attractive option for experienced operators who are skilled in this technique, particularly in cases of anatomic variations of the radial artery or weak radial pulse [2]. Anatomical dissections and radionucleotide flow studies of the ulnar and radial arteries at the wrist failed to demonstrate any difference between the anatomical dimensions of these vessels, but the radial artery was shown to have a statistically greater blood flow compared with the ulnar artery [3]. The possible methods of reaching the coronary vasculature using a percutaneous technique are limitless: radial, femoral, brachial, ulnar, subclavian, and axillary arteries and even direct puncture of the aorta from a translumbar approach, have been utilized in the past [4]. During 1989 till 1999, percutaneous radial artery approach started to be applied by cardiology interventionists. There was a considerable amount of articles that discussed about the conversion to predominantly radial access and its results [5]. Terashima et al. [6] were the first who recorded the feasibility of trans-ulnar approach for diagnostic catheterization of coronary arteries about two decade ago, and their study was followed by limited numbers of investigations later [5]. The ulnar and radial arteries are usually similar in size; however, anatomical variation may exist so that one vessel is larger. Ulnar cardiac catheterization may be associated with complications such as nerve injury causing paresthesia, hematoma formation resulting in nerve compression, digital ischemia, pseudoaneurysm formation, arteriovenous fistula, arterial dissection, arterial occlusion (symptomatic or asymptomatic), arterial spasm, arterial perforation, and access site pain [7]. Vasospasm is less likely to happen than in radial procedures given less alpha receptors in the ulnar artery [8].

## Methods

This study is a prospective single-center comparative study conducted at a single Egyptian cardiology center over a period of 18 months as a preliminary experience for operators experienced in radial approach with no/minimal trans-ulnar experience. At the end, we included 120 patients who continued to do Doppler study coming for coronary CA or PCI at our catheterization laboratory (Cath lab); patients were distributed randomly for each vascular access; procedures were performed by our staff members; and patients fell in two groups as follow: 80 patients in the radial group and 40 patients in the ulnar group. Ulnar arterial access technique is similar to the radial approach: After infiltration of a local anesthetic, arterial puncture was achieved by palpation of the site of maximal pulse prominence, (hyperextension of the wrist will often accentuate the ulnar arterial pulsation). We started the arterial puncture on the lateral side of the ulnar artery to reduce pain and spasm approximately 0.5 to 3 cm proximal to the flexor crease skinfold along the axis with the most powerful pulsation of the artery. The needle was inserted at a 45°-to-60° angle along the vessel axis and from lateral to medial, avoiding the ulnar nerve (Fernandez R et al., 2018). Using 6F Kits used routinely for radial sheaths (SCW-TIS, England & Callisto, Netherlands) the Seldinger technique was applied by passing a 0.021-in hydrophilic guidewire through the needle and after removing the needle, passing a 6-Fr hydrophilic sheath over the guidewire; vasodilators nitroglycerin (100 μg) and verapamil (2.5 mg) and heparin (50–70 IU/kg, up to 5000 U) were administered intra-arterially. In our study, 2 cases of complex bifurcational lesions were operated successfully using a 7-Fr sheathless catheter via ulnar artery approach (BAT technique) after sheath exchange.

### Inclusion criteria

Patients aged > 18 years who were admitted for coronary angiography with or without intervention were included in the study.

### Exclusion criteria

The following were excluded from the study:

1. Patients with cardiogenic shock or pulmonary edema
2. Patients who have done or prepared for coronary artery bypass grafting (CABG) using radial grafts
3. Chronic renal failure patients with arteriovenous fistula or those patients who have the potential for having arteriovenous fistula

### Patients 

All patients were subjected to the following;

1. Comprehensive history taking: Patients provided details of their demographic and social characteristics and history of current or previous medications.
2. Basic clinical examination
3. Pre and post procedure 12-lead ECG
4. Echocardiography before the procedure
5. Procedural success, procedural time, and fluoroscopy time were recorded.
6. All patients were examined carefully immediately after the procedure and before discharge to assess any complications.
7. Doppler evaluation to both radial and ulnar arteries a day after the procedure was done to all cases: patency of both radial and ulnar arteries and internal diameter of both arteries at the level of wrist; all were recoded.

### The primary outcomes of interest are clinical and procedural

The primary clinical outcome of this study was a combined endpoint of access site bleeding and access site non-bleeding complications.

1. *Access site bleeding:* small hematoma or large hematoma (documented as more than 5 cm)
2. Other vascular access site complications:

-Surgical repair or intervention on the access site

-Pseudoaneurysm

-Arterial spasm

-Arterial occlusion

-Atrioventricular (AV) fistula

-The primary procedural outcomes are as follow:

*-Procedural success* was done to assess incidence of crossover.

*-Fluoroscopy time* was recorded (in minutes).

*-Procedural time* was recorded (in minutes).

## Statistical methodology

The data were analyzed using SPSS v. 25 (Statistical Package for Social Science) for Windows.

The description of variables are as follows:

- Description of quantitative variables: mean and standard deviation (SD)
- Description of qualitative variables: numbers (No.) and percentages (%)
- Comparison between both groups regarding categorical data using the Chi-square test
- Comparison between both groups regarding scale data done using the independent *t* test
- The significance results were assessed in the form of *P* value:
- Non-significant when *P* value > 0.05
- Significant when *P* value ≤ 0.05
- Highly significant when *P* value ≤ 0.001

## Results

This study is a prospective single-center comparative study. Our study included 120 patients coming for CA or PCI (including patients planned for PCI and ad hoc PCI as ST elevation myocardial infarction (STEMI) patients); patients were distributed randomly for each vascular access, the procedures were performed by our staff members, and the primary access was as follow: 80 patients in the radial group and 40 patients in the ulnar group.

Table 1 showed that there were no statistically significant differences between both groups regarding their age (years; 57.1 ± 7.5 in the ulnar and 56.3 ± 7.1 in the radial groups) (*P* = 0.508), gender (males were 80% in the ulnar and 78.8% in the radial groups) (*P* = 0.756), smoking that was the predominant risk factor in both groups (77.5% in the ulnar compared with 73.7% in the radial groups) (*P* = 0.421), followed by dyslipidemia (65% in the ulnar and 68.7% in the radial groups) (*P* = 0.653), hypertension (60% in the ulnar compared with 58.7% in the radial group) (*P* = 0.211), and finally diabetes (50% in the ulnar and 53.7% in the radial groups) (*P* = 0.345).

**Table 1:** Demographic data of the whole study population

Demographic data	Ulnar group 40 patients (100%)	Radial group 80 patients (100%)	*P* value
Gender			
Male	32 (80%)	63 (78.8%)	0.756
Female	8 (20%)	17 (21.2%)	
Age (years)			
Mean ± SD	57.1 ± 7.5	56.3 ± 7.1	0.508
Risk factors			
DM	20 (50%)	43 (53.7%)	0.345
HTN	24 (60%)	47 (58.7%)	0.211
Smoking	31 (77.5%)	59 (73.7%)	0.421
Dyslipidemia	26 (65%)	55 (68.7%)	0.653

*P* value > 0.05, non-significant

Table 2 shows that 52.5% of cases in the ulnar group had PCI, and 47.5% had CA compared with the radial group: 48.7% PCI and 51.3% CA with no statistically significant difference between both groups (*P* = 0.699). PCI subgroups included patients planned for PCI and ad hoc PCI as in STEMI patients.

**Table 2:** Catheterization data of the whole study population

Catheterization data	Ulnar group 40 (100%)	Radial group 80 (100%)	*P* value
Procedure type			
CA	19 (47.5%)	41 (51.3%)	0.699
PCI	21 (52.5%)	39 (48.7%)	
Procedural urgency			
Elective	37 (92.5%)	78 (97.5%)	0.331
Emergency	3 (7.5%)	2 (2.5%)	
Procedure success			
Succeeded	33 (82.5%)	75 (93.7%)	0.101
Crossover	7 (17.5%)	5 (6.3%)	
Causes of crossover			
Tortuosity	0	1/5 (20%)	0.236
Spasm	0	4/5 (80%)	**0.005**
Failure to puncture	7/7 (100%)	0	**<0.001**
2ry access			
Radial artery	6 (15%)	–	
Femoral artery	1 (2.5%)	4 (5%)	0.999
Ulnar artery	–	1 (1.25%)	

*P* value > 0.05; non-significant

Regarding procedural urgency, the majority of cases underwent elective procedures; five cases of acute STEMI underwent successful primary PCI (three cases via ulnar approach and two via radial approach with no statistically significant difference between both groups) (*P* = 0.331).

In the ulnar group, we had to crossover to other access in seven cases compared with the five cases in the radial group, with no statistically significant difference between both groups (*P* = 0.101). Failure of puncture was the only cause of crossover in the ulnar group (highly significant difference; *P* < 0.001), while in the radial group, four cases showed severe radial artery spasm (*P* = 0.005). Radial artery tortuosity hindered the procedure in the fifth patient.

In failed cases of the primary approach, the protocol was to try the homolateral access if feasible. In failed ulnar cases, we succeeded to cross over to homolateral radial access in six patients and to femoral access in one case (weak radial pulsations in this case), while in failed radial cases, we had to shift to femoral access in four cases (as these cases had severe arm pain due to severe radial spasm so ulnar approach was not possible) and to homolateral ulnar access in the fifth patient.

In Table 3, the mean fluoroscopy time in the ulnar group was 5.6 ± 1.9 min in CA and 12.4 ± 2.6 min in PCI, compared with 5.3 ± 2.1 min in CA and 11.9 ± 2.3 min in PCI in the radial group, but these differences were all statistically not significant (*P* > 0.05). While the procedure time of CA and PCI of the ulnar group was significantly higher than that of the radial group (*P* values = 0.011 and 0.034, respectively), the mean procedure time in the ulnar group was 22.6 ± 2.6 min in CA and 36.1 ± 4.1 min in PCI compared with 21.2 ± 2.9 min in CA and 34.2 ± 4.8 min for PCI in the radial group.

**Table 3:** Comparison regarding the fluoroscopy and procedure times

Procedural data	Ulnar group 40 (100%)	Radial group 80 (100%)	*P* value
Fluoroscopy time (min) (Mean ± SD)			
CA	5.6 ± 1.9	5.3 ± 2.1	0.448
PCI	12.4 ± 2.6	11.9 ± 2.3	0.284
Procedure time (min)			
CA	22.6 ± 2.6	21.2 ± 2.9	**0.011**
PCI	36.1 ± 4.1	34.2 ± 4.8	**0.034**

*P* value > 0.05; non-significant

Table 4 showed that there were no statistically significant differences between both groups regarding all types of complications (*P* value > 0.05), three cases in the ulnar group (7.5%) showed ulnar artery occlusion after the procedure documented with post procedural duplex compared with two cases in the radial group (2.5%) (*P* value = 0.647). While regarding arterial spasm, our study showed that four cases in the radial group (5%) developed persistent spasm compared with one case of transient spasm in the ulnar group (2.5%) with no statistically significant difference (*P* value = 0.343). Minor hematoma occurred in the same percentage of 2.5%: 2 cases in the radial group and 1 case in the ulnar group (*P* value = 0.999).

**Table 4:** Distribution of complications in both groups:

Access site complications	Ulnar group 40 (100%)	Radial group 80 (100%)	*P* value
Arterial occlusion	3 (7.5%)	2 (2.5%)	0.647
Arterial spasm	1 (2.5%)	4 (5%)	0.343
Major bleeding	0%	0%	
Minor hematoma	1 (2.5%)	2 (2.5%)	0.999
Hematoma > 5 cm	0%	0%	
Pseudoaneurysm	0%	0%	
AV fistula	0%	0%	

*P* value > 0.05, non-significant

Table 5 showed that there was no statistically significant difference between both groups regarding the mean internal diameter of radial and ulnar arteries at the level of the wrist: mean caliber of ulnar artery was 2.45 ± 0.38 slightly larger than the radial artery 2.33 ± 0.38 (*P* value = 0.105). Regarding arterial occlusion, three cases in the ulnar group developed post procedural arterial occlusion compared with two cases in the radial group, but this difference was not statistically significant (*P* value = 0.196).

**Table 5:** Ultrasonographic data of the whole study population

Doppler data	Ulnar group 40 (100%)	Radial group 80 (100%)	*P* value
Caliber (mm)			
*Mean ± SD* *Median (IQR)*	2.45 ± 0.38 2.4 (2.13–2.70)	2.33 ± 0.38 2.3 (2.–2.5)	0.105
Patency			
Patent Occluded	37 (92.5%) 3 (7.5%)	78 (97.5%) 2 (2.5%)	0.196

*P* value > 0.05, non-significant

## Discussion

The trans-ulnar approach reduces the need for crossover to the femoral route. Hahalis et al. [9] concluded that the trans-ulnar strategy is inferior to trans-radial strategy because of high crossover rates in the trans-ulnar arm. While Gokhroo et al. [10] showed that when trans-ulnar interventions were performed by operators who were default radial operators with experience of at least 50 ulnar artery cannulations, trans-ulnar approach was non-inferior to trans-radial arm for patients undergoing coronary angiographies.

In this study, the aim was to prove whether experienced trans-radial but naive trans-ulnar operators (no/minimal trans-ulnar experience as operators) will have comparable success and complication rates, because of the deep location and weak (but definite) palpability of the ulnar artery. In this study, the ulnar approach was associated with higher crossover rate than the radial approach but without statistically significant difference; mostly due to primitive experience: 7 cases of patients randomized to the ulnar arm (17.5%) compared with 5 cases in the trans-radial arm (6.3%). In the ulnar group, failed cases crossover to the homolateral radial artery which succeeded in six cases and to the femoral artery in the seventh case due to weak radial pulse. All patients randomized for trans-radial access operators succeeded in arterial cannulation; the reason for trans-radial failure in four cases was persistent radial artery spasm in spite of using nitroglycerin and verapamil before passing the catheters with crossover to the transfemoral approach due to severe arm pain and possibility of other arm spasm. In the fifth patient, there was radial artery tortuosity though use of hydrophilic 0.035-in Terumo wire but with marked arterial kinking which halted wire progression, and there was no pain, so crossover was done to a successful homolateral ulnar approach. Previous research by Fernandez et al. [11] stated that homolateral ulnar artery should be accessed when the radial artery precludes catheterization due to spasm, tortuosity, or perforation, thereby eliminating the need for contralateral radial artery or femoral artery access. There has been no evidence to suggest that hand ischemia occurs in higher rates when the homolateral ulnar artery is accessed after failure to access the radial artery, as there are significant variations in the blood supply of the hand and the presence of multiple other arteries that supply the hand, including the interosseous and median arteries. Success rate to accomplish the procedure through primary access in the radial arm was 75 out of 80 patients (93.75%) with a failure rate of 6.25%; this is a higher rate of success compared to an earlier comparative study at the same center with the same operators [12] that showed 17% failure rate in the trans-radial group, reflecting the progress in operators’ learning curve together with the availability of modern radial kits. All cases with ulnar arterial puncturing succeeded to accomplish the procedure through ulnar access; weak ulnar pulse constituted the most common cause of puncture failure as the artery is deeply seated underneath the muscles. However, in few patients, we succeeded in puncturing the artery easily though with a weak ulnar pulse. On the other hand, despite finding a good palpable ulnar pulse in other patients, we could not access the artery. It is worth mentioning that 5 cases out 7 failed ulnar puncture were among the first 10 cases randomized to ulnar arm reflecting also the progress in our operators’ learning curve. These outcomes are similar to Sallam et al. [13] who stated that the main reason for ulnar access failure was the inability to puncture (17.7%).

Transient ulnar artery spasm occurred in 1 case only in the ulnar group (2.5%), while persistent radial artery spasm occurred in 4 cases (5%) in the radial group, and the radial, more than the ulnar spasm, was statistically significant.

The lower ulnar artery spasm rate is explained by the facts that ulnar artery is usually larger, straight, and has less alpha receptors than the radial artery which may make it less disposed to catheter-induced vasospasm compared with the radial artery. ([5]; Kedev et al ., 2014 [2];).

No major hematoma requiring intervention occurred in either group; minor hematoma occurred in the same percentage (2.5%): 2 cases in the radial group and 1 case in the ulnar group reflecting safety of both arm approaches [14].

Post procedural duplex in our study showed larger ulnar than radial arterial diameter with mean internal diameter of 2.45 ± 0.38 mm versus 2.33 ± 0.38 mm. respectively at the level of wrist. In a review by Beniwal et al. [15], the mean diameter was 2.358 ± 0.39 mm for ulnar arteries and 2.325 ± 0.4 mm for radial arteries. In our study; 2 cases of complex bifurcational lesions were operated successfully using 7-Fr sheathless catheter via the ulnar artery approach using (BAT technique) the larger ulnar diameter, and less tortuosity and spasm were the assumed causes for this relatively complex technique success. About 6% of the patients after trans-radial procedure develop vascular occlusion, hindering repeat procedures, if required, thus switching either to other radial or to femoral access [16]. Prior trans-radial procedure is associated with more intimal hyperplasia and reduced early graft patency if radial artery is used as graft in CABG [17]. Among our patients, arterial occlusion occurred in both groups without a statistically significant difference with three cases (7.5%) in the ulnar group versus two cases (2.5%) with developed radial artery occlusion; all were documented by post-procedural duplex. As there was no significant ischemia associated with this complication, no further action was needed. The whole five cases were among patients that underwent CA and not PCI which may be due to higher doses of unfractionated heparin (UFH) and loading dual antiplatelet therapy that prevented arterial spasm.

Strictly using the recommended dose of UFH (50–70 IU/kg, up to 5000 U) for all cases, using of pulse oximetry while occluding the radial and ulnar arteries and avoiding tight and prolonged compression during hemostasis decreased the incidence of radial and ulnar occlusion along the study. In the Dahal et al. [18] study that involved 2744 patients, cases that developed ulnar artery occlusion were 78 out of 1144 (6.8%), and cases that developed radial artery occlusion were 84 out of 967 (8.7%); in the Hahalis et al. [19] study, radial artery occlusion and ulnar artery occlusion occurred in similar frequency and in the order of 7% to 8% when evaluated early by vascular ultrasonography following coronary procedures. Although comparative reductions in access site complications were achieved, trans-ulnar access in our study consumed higher procedural and fluoroscopy times compared with trans-radial approach. The mean fluoroscopy time in our study was 5.6 ± 1.9 min in CA and 12.4 ± 2.6 min in PCI in the ulnar group, while in the radial group, it was 5.3 ± 2.1 min in CA and 11.9 ± 2.3 min in PCI (*P* = NS). The mean procedure time in our study was 22.6 ± 2.6 min in CA and 36.1 ± 4.1 min in PCI in the ulnar group, while in the radial group, it was 21.2 ± 2.9 min in CA and 34.2 ± 4.8 min in PCI. This higher procedure time of CA and PCI in the ulnar group was significantly different (*P* value = 0.011 and 0.034, respectively). Longer procedural time in the ulnar than radial approach seems reproducible as Hahalis et al. [9] had total procedure time of 19 min (11–30) in the radial group and 24 min (15–40) in the ulnar group (*P* = 0.001), and in more recent studies [20], the mean procedure time of trans-ulnar approach was (21 ± 11 min) slightly longer than trans-radial approach (20 ± 8 min).

## Conclusion

Trans-ulnar access in our patients is a safe and practical approach for coronary angiography or angioplasty by operators experienced in radial access, without major complications, increasing the chance of success with forearm access and reduces the need for crossover to the femoral route. It can be used as the default route when a radial artery is not useful for the catheterization like prior CABG or CRF patients and to preserve the radial artery as an arterial graft in patients scheduled for CABG.

## Study limitations

Following limitations in the current study need to be addressed:

(a) Study operators were default radialists with no/minimal trans-ulnar experience, and this was the study hypothesis.

(b) Delayed vessel occlusion due to intimal injury and hyperplasia was not addressed.

(c) This was a single-center study enrolling a small number of patients.

## Data Availability

All data generated or analyzed during this study are included in this published article.

## Abbreviations

TRA: Trans-radial approach
CA: Coronary angiography
Cath lab: Cathererization laboratory
PCI: Percutaneous coronary intervention
CAD: Coronary artery disease
ECG: Electrocardiogram
CRF: Chronic renal failure
STEMI: ST elevation myocardial infarction
TIMI: Thrombolysis in Myocardial Infarction
CABG: Coronary artery bypass grafting
Hb: Hemoglobin
AV: Atrioventricular
DM: Diabetes mellitus
HTN: Hypertension
BAT: Balloon-assisted tracking
UFH: Unfractionated heparin
SD: Standard deviation
Fr: French (unit for catheter diameter)
